# The ubiquitin-conjugating enzyme UBE2O modulates c-Maf stability and induces myeloma cell apoptosis

**DOI:** 10.1186/s13045-017-0499-7

**Published:** 2017-07-03

**Authors:** Yujia Xu, Zubin Zhang, Jie Li, Jiefei Tong, Biyin Cao, Paul Taylor, Xiaowen Tang, Depei Wu, Michael F. Moran, Yuanying Zeng, Xinliang Mao

**Affiliations:** 1 Jiangsu Key Laboratory for Translational Research and Therapeutics of Neuro-Psycho- Diseases, Department of Pharmacology, College of Pharmaceutical Sciences, Soochow University, 199 Ren Ai Road, Suzhou Industrial Park, Suzhou, 215123 Jiangsu People’s Republic of China; 2 Program in Molecular Structure and Function, The Hospital for Sick Children, Department of Molecular Genetics, University of Toronto, Toronto, M5G 0A4 Canada; 3 Department of Hematology, The First Affiliated Hospital of Soochow University, Suzhou, China; 4 Department of Oncology, Suzhou Municipal Hospital East Campus, Suzhou, 215100 People’s Republic of China; 5 Key Laboratory of Protein Modification and Degradation, School of Basic Medical Sciences, Affiliated Cancer Hospital & Institute of Guangzhou Medical University, Guangzhou, 511436 China

**Keywords:** c-Maf, UBE2O, Ubiquitin proteasome pathway, Multiple myeloma

## Abstract

**Background:**

UBE2O is proposed as a ubiquitin-conjugating enzyme, but its function was largely unknown.

**Methods:**

Mass spectrometry was applied to identify c-Maf ubiquitination-associated proteins. Immunoprecipitation was applied for c-Maf and UBE2O interaction. Immunoblotting was used for Maf protein stability. Luciferase assay was used for c-Maf transcriptional activity. Lentiviral infections were applied for UBE2O function in multiple myeloma (MM) cells. Flow cytometry and nude mice xenografts were applied for MM cell apoptosis and tumor growth assay, respectively.

**Results:**

UBE2O was found to interact with c-Maf, a critical transcription factor in MM, by the affinity purification/tandem mass spectrometry assay and co-immunoprecipitation assays. Subsequent studies showed that UBE2O mediated c-Maf polyubiquitination and degradation. Moreover, UBE2O downregulated the transcriptional activity of c-Maf and the expression of cyclin D2, a typical gene modulated by c-Maf. DNA microarray revealed that UBE2O was expressed in normal bone marrow cells but downregulated in MGUS, smoldering MM and MM cells, which was confirmed by RT-PCR in primary MM cells, suggesting its potential role in myeloma pathophysiology. When UBE2O was restored, c-Maf protein in MM cells was significantly decreased and MM cells underwent apoptosis. Furthermore, the human MM xenograft in nude mice showed that re-expression of UBE2O delayed the growth of myeloma xenografts in nude mice in association with c-Maf downregulation and activation of the apoptotic pathway.

**Conclusions:**

UBE2O mediates c-Maf polyubiquitination and degradation, induces MM cell apoptosis, and suppresses myeloma tumor growth, which provides a novel insight in understanding myelomagenesis and UBE2O biology.

## Background

The ubiquitin-conjugating enzymes (E2s) transfer activated ubiquitin molecules to a ubiquitin ligase (E3) or directly mediate substrate ubiquitination. Therefore, the E2 enzymes are critical for protein ubiquitination and stability. There are 35 E2s, of which most are small proteins with molecular weights from 14 to 35 kDa, and these E2s fail to exhibit intrinsic affinity for physiological substrates [1]; however, there is an unusually large E2 called E2-230K or UBE2O that can ubiquitinate proteins in an E3-independent manner [1]. UBE2O is ubiquitously expressed and has been proposed to play a role in erythroid differentiation [2, 3]. Recently, it is found to mediate monoubiquitination of SMAD6 [4] and polyubiquitination of BAP1 [5] and AMPKα2 [6]. UBE2O also inhibits TRAF6 K63-polyubiquitination [7]. Therefore, UBE2O might exhibit various functions upon the contexts.

Multiple myeloma (MM) is an incurable malignancy derived from plasma cells. Although the detailed mechanisms are not fully understood, the Maf family transcription factors raise high attention [8]. This family is comprised of seven members of which c-Maf, MafA, and MafB belong to the large Maf subfamily because these transcription factors contain the complete structure and functional domains including the DNA-binding domain and the transcription activation domain [8]. All c-Maf, MafA, and MafB are found to be highly overexpressed in MM cells in association with chromosomal translocations and other unknown mechanisms [9]. Notably, c-Maf is reported in more than 50% of MM cell lines and primary MM patient samples [10]. Overexpression of c-Maf has been regarded as a key factor in MM pathophysiology because interference with c-Maf blocks MM tumor growth in immunodeficient mice [10], while c-Maf-transgenic mice develop MM-like symptoms at old age around 50–60 weeks old [11]. Therefore, c-Maf is proposed as a target for MM therapy. Our recent study found that c-Maf undergoes degradation via the ubiquitin-proteasome pathway under the direction of ubiquitin ligase HERC4 [12]. However, its ubiquitin-conjugating enzyme is yet to know.

In the study of c-Maf ubiquitination-associated enzymes by the affinity purification-coupled mass spectrometry (AP/MS) strategy, UBE2O was identified in the c-Maf co-immunoprecipitates and it interacts with c-Maf protein and mediates c-Maf ubiquitination and degradation. More importantly, UBE2O induces c-Maf expressing MM cell apoptosis and delays MM tumor growth in mice.

## Methods

### Primary bone marrow cells

Primary bone marrow species were collected from the Department of Hematology, the First Affiliated Hospital of Soochow University. The use of primary bone marrow cells was approved by the Review Board and Ethical Committee of Soochow University, and each patient provided written informed consent to donate 2–5 ml of bone marrow for this study after diagnostic and clinical procedures in accordance with the Declaration of Helsinki. Mono-nuclear cells were isolated by Lympholyte® Cell Separation (Cedarlane, Canada) [13].

### Constructs

c-Maf and MafA were cloned as described previously [14]. UBE2O cDNA (Genebank accession No. BC051868.2) was obtained from the SPARC BioCentre, The Hospital for Sick Children, Toronto, Canada.

### Lentiviral UBE2O

To generate UBE2O-expressing lentivirus, the cDNA fragment of UBE2O was generated by using the following primers: 5′-TCGAGCTCAAGCTTATGACCTCAGCCGACGTGATG-3′ (forward) and 5′-CTCACCATGACCCATGACCGGTGGATCCTCCTTGTCCTCTGTGCACTCCG-3′ (reverse) and inserted into pLVX-AcGFP lentiviral vector (Clontech) within the EcoRI and BamHI sites. The specific protocol for packaging of the virus and preparation of lentiviral particles were described previously [12].

### Affinity purification-coupled mass spectrometry (AP/MS)

The AP/MS process and data analysis were performed as described previously [12].

### Immunoprecipitation (IP)

HEK293T cells were transfected with c-Maf and UBE2O plasmids for 24–48 h followed by immunoprecipitation as described as previously [15] using specific primary antibodies as needed followed by incubation with 40 μl of a 50% slurry of protein A+G agarose beads with gentle rotation at 4 °C for 2 h. Agarose beads were collected and washed five times with the lysis buffer [12], followed by re-suspension in 20 μl of 2× SDS loading buffer. Samples were then boiled before being subjected to fractionation on sodium dodecyl sulfate poly-acrylamide gel electrophoresis (SDS-PAGE) and immunoblotting (IB) analysis.

### In-tube ubiquitination assay

This assay was adapted from a previous report [16]. Briefly, HA-c-Maf and Flag-UBE2O plasmids were transfected into HEK293T cells, respectively. Forty-eight hours later, cells were treated with MG132 for 2 h, followed by cell lysate preparation. To enrich and purify c-Maf and UBE2O proteins, individual cell lysates were subjected to immunoprecipitation with HA- (for c-Maf) or Flag- (for UBE2O) antibody-conjugating agarose beads, respectively, at 4 °C for overnight. After that, the beads were washed four times with an immunoprecipitation lysis buffer, twice with 1× ubiquitin reaction buffer (Boston Biochem, Boston, MA) and then re-suspended in 20 μl of 1× ubiquitin reaction buffer containing 200 ng of recombinant E1, 250 ng of recombinant UbcH5c, 10 μg of ubiquitin, 0.5 mM ATP, and 1× Energy Restoration System (Boston Biochem, Boston, MA). The reaction was carried out at 30 °C for 2 h and then terminated by boiling in the 2× SDS loading buffer. Ubiquitinated products were resolved by SDS-PAGE and detected by immunoblotting analysis.

### Immunoblotting (IB) analyses and cycloheximide (CHX) chase analysis

All the IB and CHX chase assays were performed as described previously [15].

### Cell proliferation assay

MM cells were infected with lentiviral UBE2O for 1 - 7 d before being subjected to MTT (3-(4,5-dimethylthiazol-2-yl)-2,5-diphenyltetrazolium bromide) assay as described previously [17, 18].

### Cell cycle analysis

MM cells were infected with lentiviral UBE2O for 48 h; cells were collected and treated with cold 70% ethanol and stained with propidium iodide (PI). Cell cycle was analyzed on a cytometer (FACSCalibur; BD Biosciences, San Jose, CA) as described previously [19].

### Cell apoptosis by cytometry

MM cells were infected with lentiviral UBE2O at various periods; cells were then stained with 7-ADD and Annexin V-PE (MultiSciences BiotechCo., Ltd., Hangzhou, China) followed by analysis on a cytometer as described previously [20].

### Luciferase assay

The luciferase reporter plasmid pGL4-CCND2-Luc was constructed as described previously [15]. The DNA sequence of CCND2 promoter contained a c-Maf recognition element (MARE) which could be recognized by c-Maf protein [10, 21]. To examine the effect of UBE2O on c-Maf biological function, HEK293T cells were co-transfected with pGL4-CCND2, c-Maf, and UBE2O, and a β-gal expression vector (100 ng). Luciferase and β-gal expression were measured 36 h after transfection according to the manufacturer’s protocols (Promega, Madison, WI, USA). Firefly luciferase activity was normalized to β-gal expression for each sample [15]. All transfection experiments were performed in duplicates.

### GEO dataset analyses

The DNA microarray dataset from primary MM patients and healthy donors was retrieved from Gene Expression Omnibus (GEO) databases (http://www.ncbi.nlm.nih.gov/gds) [22]. Log_2_(UBE2O mRNA level) was reported.

### Reverse transcription polymerase chain reaction (RT-PCR)

Total RNA was extracted using Trizol® (Transgene, Beijing, China). RNA (2.5 μg) was reversely transcribed using a Superscript^TM^-III kit (Invitrogen) according to the manufacturer’s instruction. PCR amplification was carried out in 25 μL of PCR reaction mixture containing 10 mM Tris-HCl (pH 8.3), 50 mM KCl, 2 mM MgCl_2_, 20 pmol of each primer set, two units of Taq DNA polymerase (Transgene, Beijing, China), 0.2 mM dNTPs, and 2 μL cDNA. The primers for UBE2O were 5′-ACATCCGCTCCAACGAC-3′ and 5′-GCTGGTGCTGCCTTCTAC-3′, and the primers for GAPDH were 5′-CCAGCCGAGCCACATCGCTC-3′ and 5′-ATGAGCCCCAGCCTTCTCCAT-3′. PCR products were visualized by ethidium bromide staining after electrophoresis on 1.5% agarose gels. The optical densities of the genes were quantified using the ImageJ software (National Institutes of Health, Bethesda, MD) [19].

### Xenografts in nude mice

LP1 cells (2 × 10^7^) were s.c. inoculated into the right flanks of nude mice (The SLAC Experimental Animal Co., Shanghai, China). When the tumors were palpable, 10 μg of UBE2O plasmids or empty vectors in 100 μl of In Vivo-jetPEI® Delivery Reagent (*N*/*P* = 6) (Polyplus-transfection Inc., New York, USA) [23, 24] were injected into tumors twice a week for continued 3 weeks [17]. Tumor sizes and mice body weights were monitored every other or 3 days. This xenograft study was approved by the Review Board of Animal Ethics of Soochow University. At the end of the experiment, all tumor species were excised for immunoblotting analysis against specific antibodies as indicated.

### Statistical analysis

Student’s *t* test was used to calculate *P* values for differences. Differences were considered significant at *P* < 0.05.

## Results

### UBE2O interacts with c-Maf

**Fig. 1:**
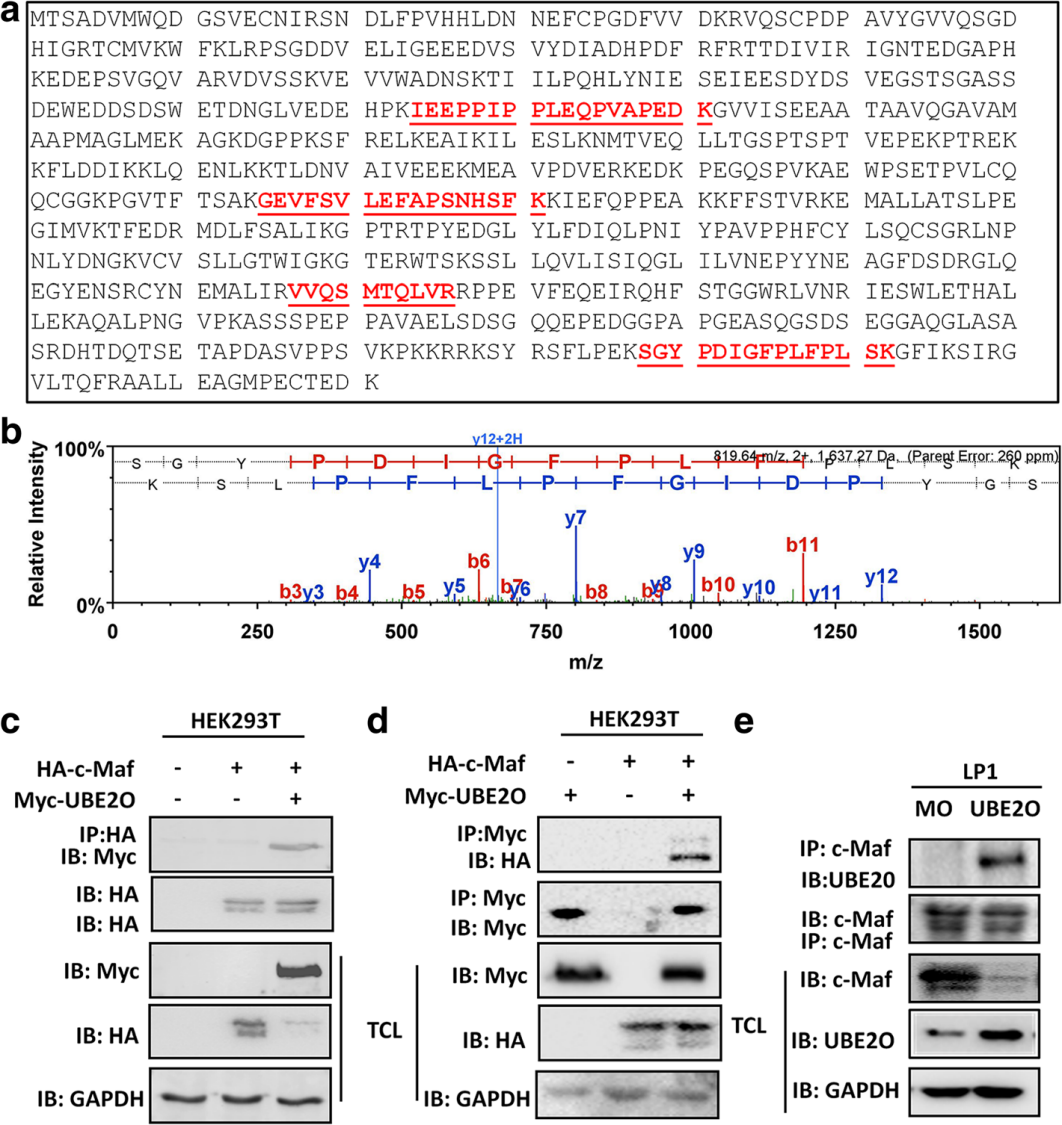
UBE2O interacts with c-Maf protein. **a** UBE2O protein was identified by MS in the c-Maf co-immunoprecipitated complex. The unique peptides found in the c-Maf IPs were underlined and highlighted in *red*. **b** A typical MS spectrum (SGYPDIGFPLFPLSK) of UBE2O. **c**, **d** The HA-tagged c-Maf plasmid and the Myc-tagged UBE2O plasmid were co-transfected into HEK293T cells for 48 h. Lysates were precipitated with an anti-HA or anti-Myc antibody followed by immunoblotting with specific antibodies as indicated. Total cell lysates (TCL) were subjected to immunoblotting assay as control. **e** Myeloma cell line LP1 was infected with lentiviral UBE2O for 72 h, followed by cell lysate preparation and IP assay

To find out specific enzymes responsible for c-Maf ubiquitination, a LC/MS/MS assay was performed after affinity purification with a c-Maf specific antibody [12]. The LC/MS/MS identified 104 proteins specifically associated with c-Maf that were enriched into two “pathways” by KEGG: the “proteasome pathway” containing 24 proteins and the “ubiquitin-mediated proteolysis” containing 6 proteins [12] from which UBE2O was found to be the only ubiquitin-conjugating enzyme. As shown in Fig. 1a, unique UBE2O peptides were identified from the co-immunoprecipitates of c-Maf protein. A typical peptide (SGYPDIGFPLFPLSK) identified by MS was shown in Fig. 1b, and specific MS data on UBE2O was presented in Table 1.

**Table 1:** The mass spectrometry data of UBE2O

Number	Peptide Sequences	Prob. (%)	Seq.Xcorr	Seq. DeltaCn	X!TANDEM	Modi.	Obs. Ms.	Act. Ma.	Charge (Z)	Δ Data
1	VVQSmTQLVR	100	3.2051642	0.22036938	1.69897	Oxidation (+16)	589.3776	1176.74	2	1.107
2	SGYPDIGFPLFPLSK	100	2.9653754	0.37091812	3.638272		819.7518	1637.49	2	0.6387
3	IEEPPIPPLEQPVAPEDK	100	2.4079683	0.5075285	2		999.8068	1997.60	2	0.5628
4	GEVFSVLEFAPSNHSFK	100	3.736957	0.2008453	1.39794		948.126	1894.24	2	0.3113

To verify the interaction between UBE2O and c-Maf, a UBE2O and a c-Maf plasmid were co-transfected into HEK293T cells, from which the lysates were co-immunoprecipitated with a specific anti-HA antibody followed by immunoblotting. As shown in Fig. 1c, UBE2O was found in the c-Maf precipitates but not in controls. To confirm this finding, a reciprocal Co-IP was performed with a specific antibody against UBE2O, followed by immunoblotting against c-Maf and the result showed that c-Maf was present in the UBE2O precipitates (Fig. 1d). To further check this finding in MM cells, lentiviral UBE2O was infected in a MM cell line LP1. In this experiment, c-Maf was used as a baitor. As shown in Fig. 1e, UBE2O was identified in the c-Maf precipitates. Therefore, these immunoprecipitation/immunoblotting assays suggested that c-Maf interacted with UBE2O.

### UBE2O mediates c-Maf polyubiquitination

**Fig. 2:**
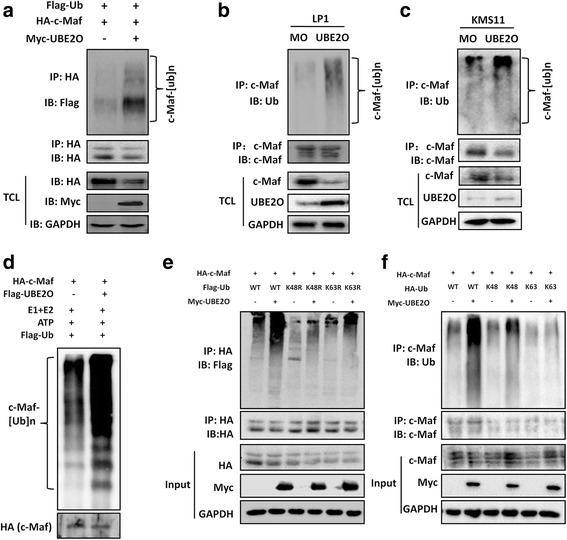
UBE2O mediates c-Maf ubiquitination independent of an E3 ligase. **a** Flag-tagged ubiquitin (Ub), HA-tagged c-Maf, and Myc-tagged UBE2O were co-transfected into HEK293T cells for 48 h. Cell lysates were precipitated with anti-Flag beads followed by immunoblotting with an anti-c-Maf antibody. Total cell lysate (TCL) was used as a control with specific antibodies as indicated. **b**, **c** Myeloma cell lines LP1 (**b**) and KMS11 (**c**) were infected with lentiviral UBE2O, respectively, for 72 h, followed by cell lysate preparation and IP assay as indicated. **d** Purified c-Maf protein was incubated with E1, UBCH5c (E2), ATP, and Ub in the presence or absence of UBE2O protein at 37 °C for 2 h. After the termination, the reaction mixture was subjected to SDS-PAGE and immunoblotting assay against c-Maf. **e**, **f** The Ub mutants were co-transfected with UBE2O and c-Maf plasmids. Twenty-four hours later, cell lysates were prepared for immuunoprecipitation and immunoblotting assay

Because UBE2O is a ubiquitin-conjugating enzyme and it has been reported to mediate ubiquitination of several proteins, we thus wondered whether UBE2O could mediate c-Maf ubiquitination given its interaction with c-Maf. To this end, Myc-UBE2O, Flag-Ub, and HA-c-Maf plasmids were co-transfected into HEK293T cells. After immunoprecipitation with an anti-Flag antibody (for Ub) followed by immunoblotting with an anti-HA antibody (for c-Maf), polyubiquitination level of c-Maf was found to be markedly increased by UBE2O (Fig. 2a). Next, we wondered c-Maf ubiquitination in MM cells in the presence of UBE2O. To this end, myeloma cell lines LP1 and KMS11 were infected with lentiviral UBE2O. Seventy-two hours later, cells were subjected to whole cell lysate preparation, followed by immunoprecipitation with a c-Maf specific antibody and immunoblotting with an anti-ubiquitin antibody. As shown in Fig. 2b, c, UBE2O markedly increased the polyubiquitination level of c-Maf in both cell lines. Because UBE2O is the only E2 that was reported to display ubiquitin ligase activity [1], we wondered whether UBE2O could mediate c-Maf polyubiquitination independent of an E3 ligase. To this end, an in-tube ubiquitination assay was performed. As shown in Fig. 2d, UBE2O markedly added ubiquitin molecules to c-Maf protein in the absence of an extra E3 ligase. Because E3 ligase is essential for the ubiquitination of a specific protein, this finding suggested that UBE2O possesses an E3 liage activity in c-Maf ubiquitination. There are various ubiquitination manners of which the most common ones are the K48-linked and the K63-linked ubiquitination, and each type of ubiquitination leads to unique functions and outcomes, and previous studies showed that UBE2O could lead to monoubiquitination [4] and K63-linked polyubiquitination [5]; we wondered which type of ubiquitination was mediated by UBE2O on c-Maf. To this end, the ubiquitin plasmids with K48R or K63R mutation were co-transfected into HEK293T cells along with c-Maf and UBE2O. The subsequent immunoprecipitation/immunoblotting assays showed that the K63R-Ub mediated c-Maf ubiquitination in the presence of UBE2O, but when K48 was mutated (K48R), c-Maf failed to be ubiquitinated by UBE2O (Fig. 2e). Consistent with this finding, K48- but not K63-Ub mediated ubiquitin conjugation to c-Maf (Fig. 2f). Therefore, UBE2O mediates the K48-linked polyubiquitination on c-Maf.

### UBE2O mediates c-Maf turnover in the proteasomes

**Fig. 3:**
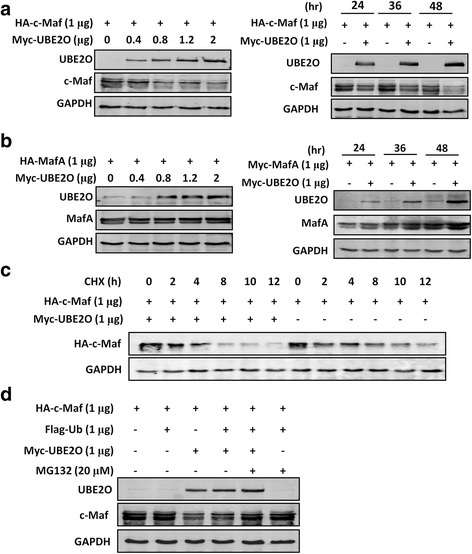
UBE2O modulates the degradation of c-Maf but not MafA. **a** HA-tagged c-Maf and Myc-tagged UBE2O plasmids were co-transfected into HEK293T cells for 48 h (*left panel*) or indicated time points (*right panel*). Whole cell lysates were subjected to immunoblotting assay against specific antibodies. GAPDH was used as an internal control. **b** HA-tagged MafA and Myc-tagged UBE2O plasmids were co-transfected into HEK293T cells for 48 h (*left panel*) or indicated time points (*right panel*). Whole cell lysates were subjected to immunoblotting assay against specific antibodies anti-HA for MafA and anti-Myc for UBE2O. GAPDH was used as an internal control. **c** HA-tagged c-Maf and Myc-tagged UBE2O were co-transfected into HEK293T cells; 24 h later, cells were treated with cycloheximide (CHX) for indicated periods. Cell lysates were subjected to immunoblotting assay against HA (c-Maf). **d** HA-c-Maf plasmids were transfected alone or together with Flag-Ub, Myc-UBE2O into HEK293T cells. Twenty-four hours later, cells were treated with 20 μM of MG132 for 4 h. Cell lysates were subjected to immunoblotting assay against indicated proteins

The K48-linked polyubiquitinaiton usually results in protein degradation. Because the above studies revealed that UBE2O mediated K48-linked polyubiquitination of c-Maf, we next investigated whether UBE2O modulated c-Maf degradation. To this end, a c-Maf plasmid was transfected into HEK293T cells along with increased Myc-UBE2O. Immunoblotting revealed c-Maf protein was decreased by UBE2O in a concentration- and time-dependent manner (Fig. 3a). However, UBE2O failed to induce degradation of MafA, another key member in the large Maf subfamily (Fig. 3b). Degradation of c-Maf by UBE2O was further demonstrated by the CHX chase assay from which UBE2O markedly shortened the time for c-Maf degradation (Fig. 3c). Moreover, UBE2O-mediated c-Maf protein degradation was abolished by MG132, a proven proteasomal inhibitor (Fig. 3d). All the above results thus led to a conclusion that UBE2O mediated c-Maf polyubiquitination and degradation in the proteasomes.

### K331 and K345 are critical for c-Maf stability mediated by UBE2O

**Fig. 4:**
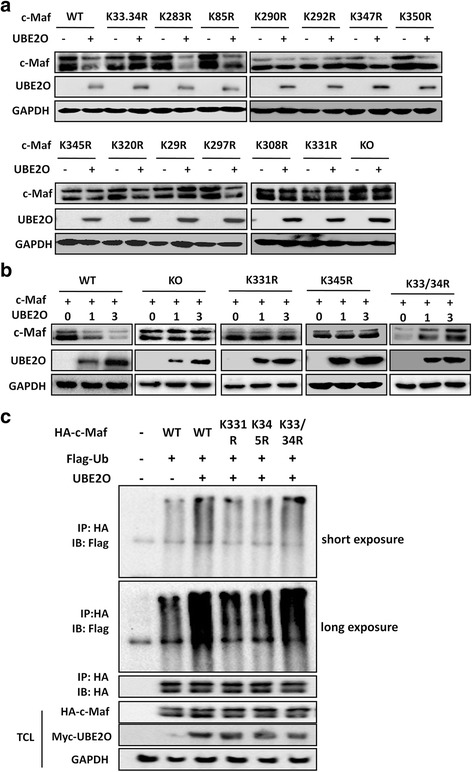
K331 and K345 are critical for UBE2O-mediated c-Maf degradation. **a** Wild-type and lysine residual mutated c-Maf plasmids were individually transfected into HEK293T cells with or without UBE2O. Twenty-four hours later, cell lysates were prepared for immunoblotting against specific antibodies as indicated. **b** The plasmids of K331R or K345R c-Maf mutants were co-transfected with increased UBE2O for 24 h. Wild-type (WT) and all lysine residual mutant (KO) c-Maf were used as positive and negative control. **c** The plasmids of K331R and K345R c-Maf mutants or control plasmids were co-transfected into HEK293T cells, respectively, with a Ub plasmid for 24 h, followed by IP and immunoblotting assays as indicated

The above results clearly demonstrated that UBE2O as a ubiquitin-conjugating enzyme mediates c-Maf ubiquitination. It reported that there are 14 lysine residues in c-Maf protein, and our previous studies showed that each lysine residue was important for c-Maf ubiquitination [12, 15]. To find out which lysine residue was important in UBE2O-mediated c-Maf ubiquitination, we generated a series of lysine (K) to arginine (R) mutants of c-Maf, followed by co-transfection with UBE2O. The measurement of c-Maf protein levels by immunoblotting assay indicated that UBE2O led to degradation of the wild-type c-Maf and most K to R mutants including K85R, K283R, K290R, K297R, K320R, K347R, and K350R (Fig. 4a). However, it failed to decrease c-Maf proteins with several mutants, including K29R, K33/34R, K308R, K331R, and K345R (Fig. 4a), which was similar to K0, the all K to R mutant (Fig. 4a) and previous studies [15]. Among these mutants, K331R and K345R were further confirmed not to mediate c-Maf degradation. As shown in Fig. 4b, UBE2O decreased the wild-type c-Maf in a concentration-dependent manner, but had no effects on the stability of the K331R, K345R, and K0 mutants. Notably, UBE2O increased the protein levels of the K33/34R c-Maf mutant (Fig. 4b) which was consistent with the results observed in a single dose of UBE2O transfection (Fig. 4a). To further confirm this finding, K331R, K345R, and K33/34 mutant plasmids were individually transfected into HEK293T cells along with UBE2O, followed by immunoprecipitation with an HA antibody (for c-Maf) and immunoblotting using a Flag antibody (for Ub). As shown in Fig. 4c, UBE2O failed to induce polyubiquitination on c-Maf with K331R or K345R mutation; in contrast, the wild-type and K33/34R c-Maf proteins were mediated polyubiquitination by UBE2O (Fig. 4c). Therefore, the K331 and K345 residues were critical for UBE2O-mediated c-Maf degradation.

### UBE2O suppresses c-Maf transcriptional activity

**Fig. 5:**
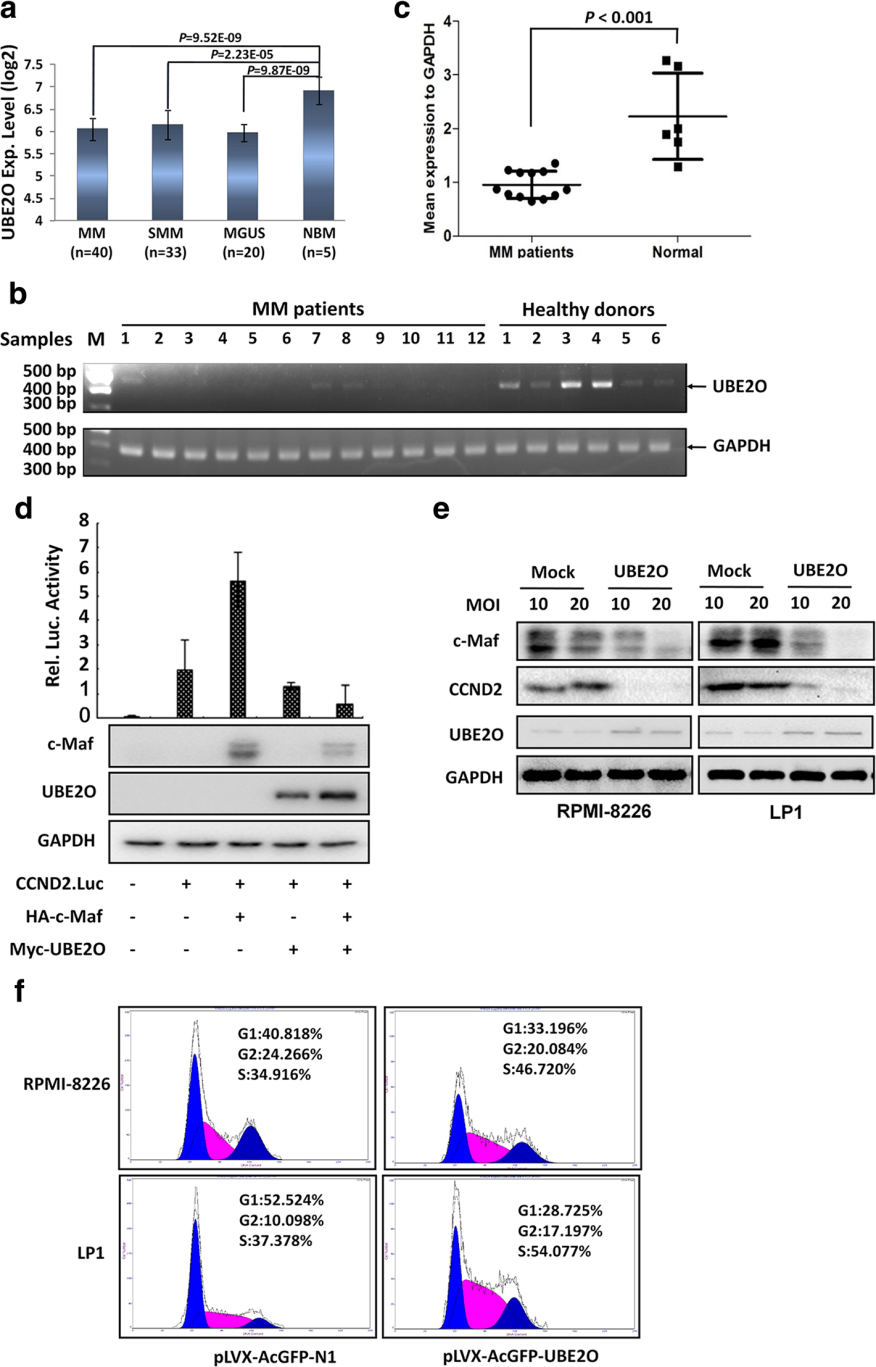
UBE2O is downregulated in MM cells and suppresses c-Maf transcriptional activity. **a** Relative UBE2O expression in the process of MM development. The mRNA expression levels of UBE2O were retrieved from a DNA microarray with 99 primary bone marrow samples including normal donors, MGUS, SMM, and MM patients as described in the “Methods” section. **b** The UBE2O mRNA levels from primary samples were analyzed by RT-PCR and agarose gel electrosphoresis. **c** Quantitative analyses of UBE2O mRNA from healthy donors and MM patients from (**b**). **d** UBE2O was co-transfected into HEK293T cells with a c-Maf plasmid and a luciferase reporter driven by a CCND2 promoter containing c-Maf recognition element (CCND2.Luc). **e** Lentiviral UBE2O was infected into MM cell lines RPMI-8226 and LP1 for 72 h. The cell lysates were then prepared for immunoblotting assay against specific antibodies as indicated. **f** RPMI-8226 and LP1 cells were infected with lentiviral UBE2O for 48 h followed by PI staining and cell cycle analysis

c-Maf is an oncogenic transcription factor that modulates the expression of several key genes including cyclin D2 involved in MM pathophysiology [10]. To explore the expression profile of UBE2O in MM cells, mRNA analysis was performed on a DNA microarray dataset from a panel of primary bone marrow samples of healthy donors, MGUS (monoclonal gammopathy of undetermined significance), SMM (smoldering MM), and MM patients [12, 22]. It showed that UBE2O was expressed in the healthy donors, but was significantly decreased in MGUS, SMM, and MM patients (Fig. 5a). To confirm this, UBE2O was measured in primary bone marrow cells from MM patients and healthy donors using RT-PCR. As shown in Fig. 5b, c, UBE2O in the bone marrow cells from MM patients were significantly lower than that from healthy donors, which was consistent with the DNA microarray assay (Fig. 5a). Therefore, UBE2O was downregulated in the process of myelomagenesis.

To further evaluate the effects of UBE2O on c-Maf activity, a luciferase reporter assay was performed after UBE2O was transfected into HEK293T cells along with pCCND2.Luci with or without c-Maf plasmids. The results showed that c-Maf markedly increased the luciferase activity under the control of CCND2 promoter containing a c-Maf recognition element (Fig. 5d) [15]. To confirm this conclusion, we next measured c-Maf and cyclin D2 levels in MM cells after MM cell lines LP1 and RPMI-8226 were infected with lentiviral UBE2O. As shown in Fig. 5e, both c-Maf and CCND2 were downregulated when UBE2O was re-expressed (Fig. 5e). However, when these MM cell lines infected with lentiviral UBE2O were subjected to cell cycle analysis, the results showed that UBE2O arrested both RPMI-8226 and LP1 cells at the S or S/M phases (Fig. 5f), which was out of expectation because cyclin D2 is a key player in control the progress of cell cycle from G1 to S phase.

### UBE2O suppresses proliferation and induces apoptosis of MM cells expressing c-Maf but not the MM cells lacking c-Maf

**Fig. 6:**
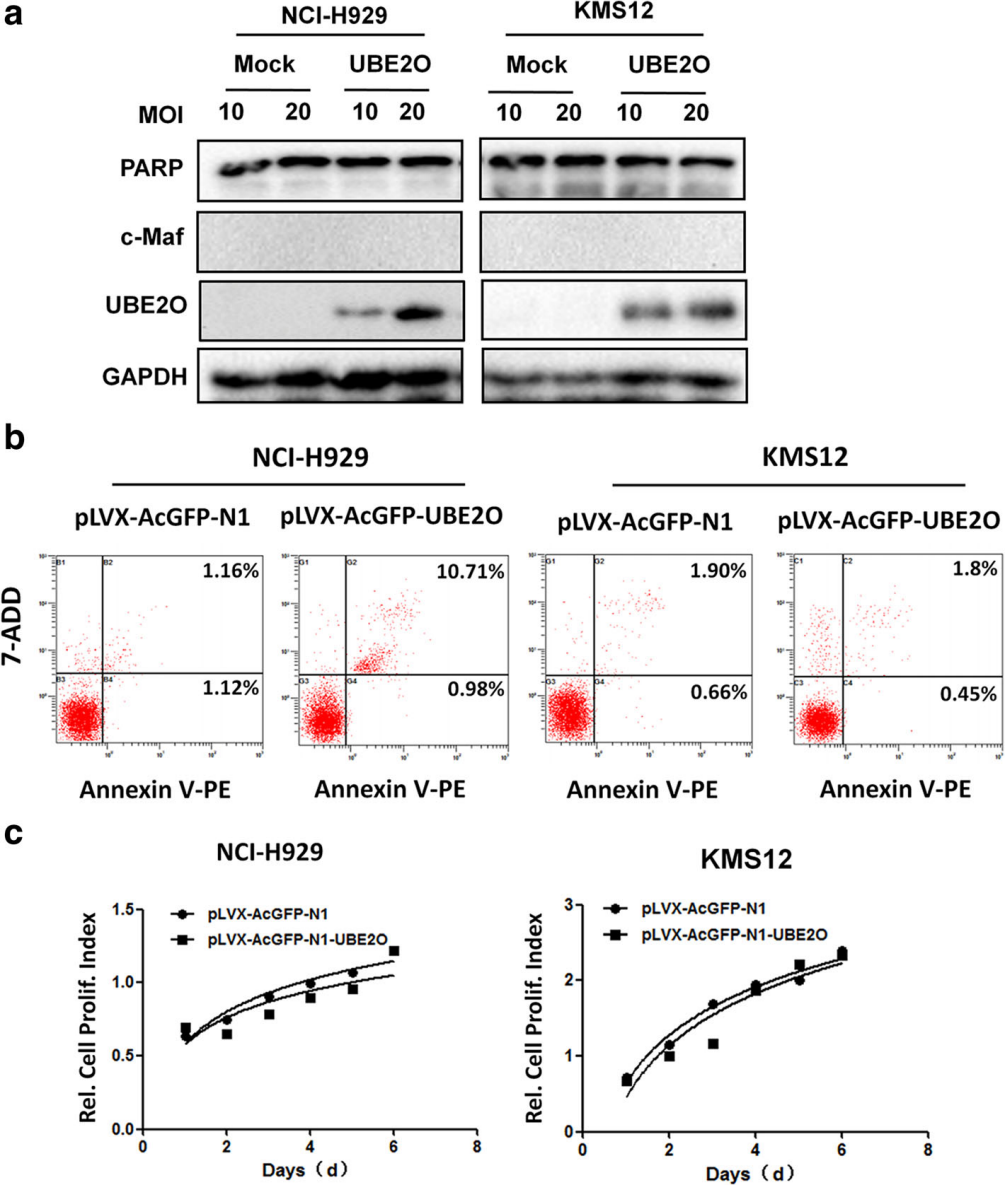
UBE2O fails to induce apoptosis of c-Maf-lacking MM cells. **a** MM cell lines NCI-H929 and KMS12 were infected with lentiviral UBE2O for 48 h, followed by immunoblotting assay against indicated antibodies. **b** NCI-H929 and KMS12 cells were infected with lentiviral UBE2O for 48 h, followed by 7-ADD and Annexin V-PE staining and flow cytometric analysis. **c** NCI-H929 and KMS12 were infected with lentiviral UBE2O for indicated periods followed by MTT assay

**Fig. 7:**
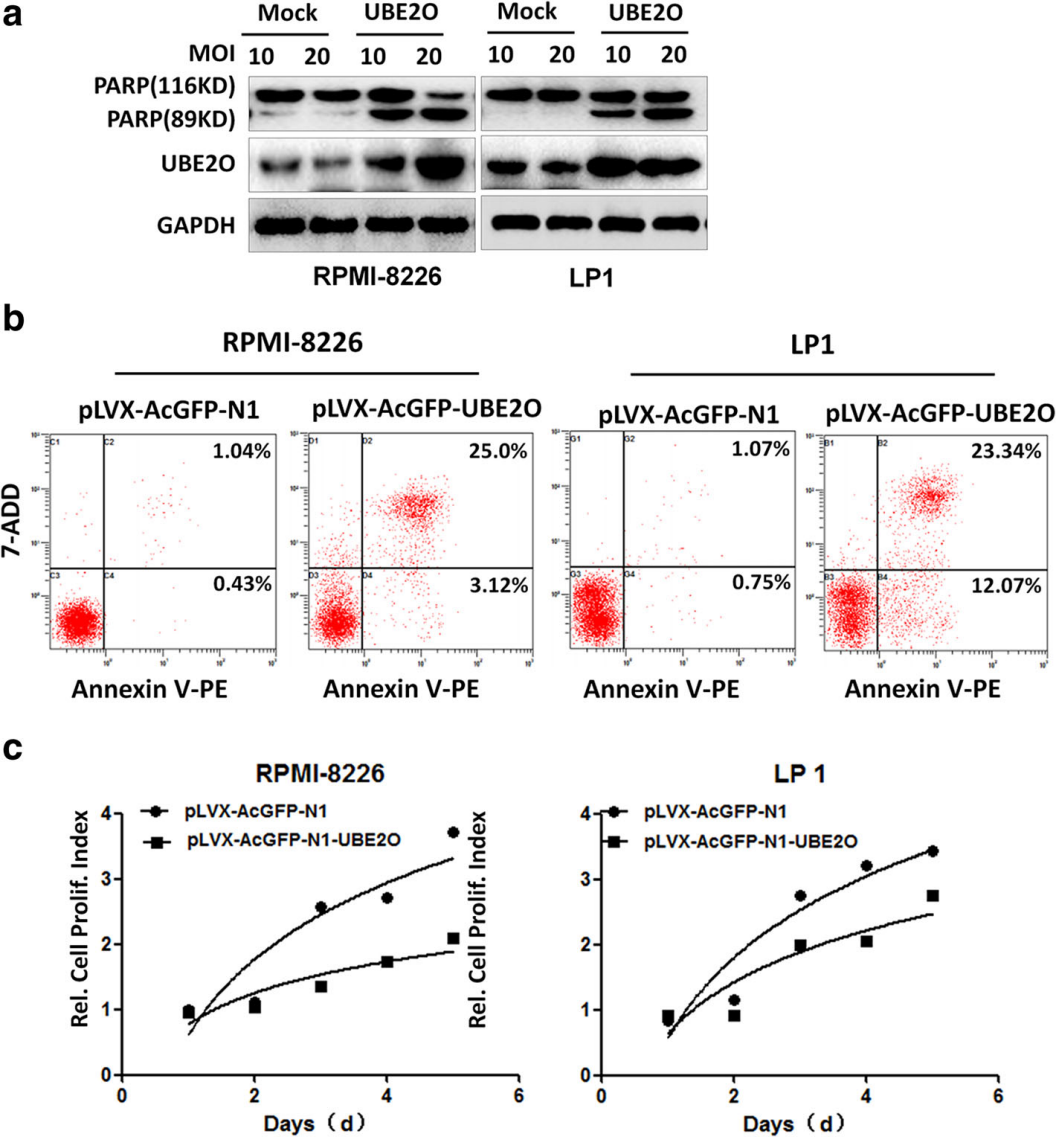
UBE2O induces apoptosis of MM cell lines expressing c-Maf. **a** RMPI-8226 and LP1 cells were infected with lentiviral UBE2O for 48 h, followed by cell lysate preparation and immunoblotting against indicated antibodies. **b** RMPI-8226 and LP1 cells were infected with lentiviral UBE2O for 48 h followed by 7-ADD and Annexin V-PE staining and flow cytometric analysis. **c** RPMI-8226 and LP1 cells were infected with lentiviral UBE2O for indicated periods, followed by MTT assay

The above studies demonstrated that UBE2O mediated c-Maf degradation and cell cycle arrest but it was downregulated in MM cells; we next evaluated the effects of UBE2O on proliferation and apoptosis in various MM cells with different levels of c-Maf. As shown in previous reports [21] and the current study, RPMI-8226 and LP1 cells expressed a high level of endogenous c-Maf (Fig. 5e), while NCI-H929 and KMS12 cells expressed a very low level of endogenous c-Maf (Fig. 6a); these cell lines were applied for this study. All cells were infected with lentiviral UBE2O followed by evaluation of cell viability and apoptosis. We first measured the cleavage level of PARP, a hallmark of apoptosis. As measurement by immunoblotting, UBE2O had no effect on PARP cleavage in NCI-H929 or KMS12 that lack c-Maf (Fig. 6a) but induced PARP cleavage in RPMI-8226 and LP1 that express c-Maf (Fig. 7a), indicating UBE2O probably induced MM cell apoptosis in association with c-Maf expression. To confirm this finding, MM cells after lentiviral UBE2O infections were subjected to apoptotic analysis by a flow cytometer after staining with Annexin V, a direct hallmark of cell apoptosis. As shown in Fig. 6b, UBE2O failed to increase the Annexin V+ fraction in NCI-H929 or KMS12 cells, but markedly raised this fraction in RPMI-8226 and LP1 cells (Fig. 7b). Because UBE2O arrested cell cycle of MM cells expressing c-Maf, we next assayed cell proliferation status after infection with lentiviral UBE2O by the MTT method. The results showed that UBE2O failed to inhibit the proliferation of NCI-H929 or KMS12 cells (Fig. 7c), but it inhibited the proliferation of RPMI-8226 and LP1 cells in a time-dependent manner (Fig. 7c). These findings were consistent with c-Maf expression levels and c-Maf degradation by UBE2O. Therefore, these results demonstrated that UBE2O induced MM cell apoptosis and suppressed MM cell proliferation in association with c-Maf expression.

### UBE2O delays myeloma tumor growth in nude mice

**Fig. 8:**
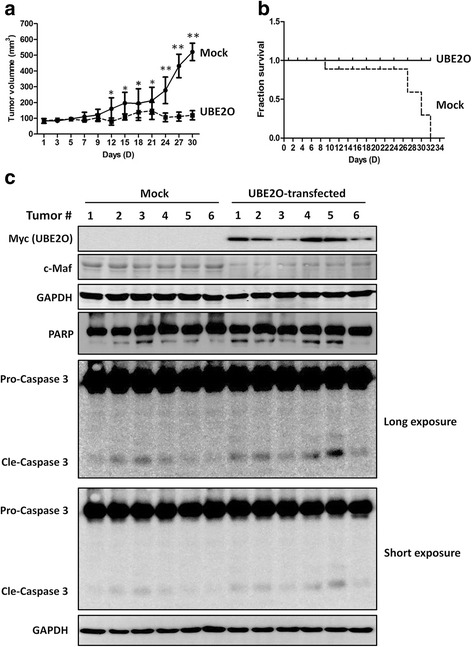
UBE2O delays MM tumor growth and prolongs the survival of mice bearing MM tumors. **a** LP1 cells were subcutaneously injected into the right flanks of female nude mice. When tumors were palpable, tumors were injected with 10 μg of UBE2O or empty vectors twice a week for continued 3 weeks. The curves of tumor sizes verse treatment days were plotted. *p < 0.05, **p < 0.01, compared with control. **b** Survival periods of nude mice were recorded. **c** Tumors tissues were applied for total protein preparation and immunoblotting assay against specific antibodies as indicated

Previous studies have demonstrated that degradation of c-Maf or interference of c-Maf leads to delayed MM tumor growth in nude mice [10, 12]; we wondered whether UBE2O generated similar effects on MM tumor growth in vivo. Myeloma xenografts with human MM cell line LP1 were established in immunodeficient nude mice, followed by intratumoral transfection of UBE2O plasmids delivered by in vivo *jetPEI* as demonstrated previously [17, 23, 24]. The results showed that re-expression of UBE2O decreased myeloma tumor growth (Fig. 8a). UBE2O also significantly extended survival time of myeloma-bearing mice. As shown in Fig. 8b, all mice carrying myeloma tumors and being transfected with control plasmids died within 32 days; in contrast, all mice survived when receiving UBE2O in an intratumoral manner. Notably, endogenous c-Maf protein in MM tumors was also decreased in the mice receiving UBE2O (Fig. 8c). To find out whether UBE2O induced apoptosis in MM tumor tissues, all tumor samples were subjected to immunoblotting assay against PARP and caspase-3. As shown in Fig. 8c, UBE2O induced markedly cleavage and activation of PARP and caspase-3, a signal of apoptosis. Therefore, UBE2O downregulated c-Maf and delayed MM tumor growth.

## Discussion

The above results demonstrated that UBE2O is a ubiquitin-conjugating enzyme that mediates c-Maf polyubiquitination in the absence of ubiquitin E3 ligases. UBE2O induces K48-linked polyubiquitinaition of c-Maf and mediates its degradation in the proteasomes. Moreover, UBE2O displays as a tumor suppressor against MM.

There are more than 35 E2s, of which most are small proteins with molecular weight from 14 to 35 kDa, and these E2s do not exhibit intrinsic affinity for physiological substrates but usually coordinate with E3s to mediate protein ubiquitination [1]. As the only large-size E2, UBE2O has been reported to modify several important proteins in an atypical ubiquitination manner, such as K63-polyubiquitination [5] and monoubiquitination [4]. Accordingly, UBE2O modulates the biological functions of its target proteins. For example, UBE2O mediates multiple mono-ubiquitination of BAP1, a nuclear localization signal, and leads to its cytoplasmic sequestration [5]. Recently, UBE2O was found to inhibit, rather than to induce, TRAF6 polyubiquition [7]. In the present study, we found that UBE2O induces c-Maf polyubiquitination. This is consistent with that finding that UBE2O mediates the K48-linked polyubiquitination of c-Maf, a typical ubiquitination manner in modulating protein stability, because when K48 was mutated, c-Maf failed to be ubiquitinated by UBE2O. Furthermore, we demonstrated that UBE2O also functions as an E3 ubiquitin ligase because it alone suffices to mediate c-Maf ubiquitination in tube in the absence of any E3 ligases. Therefore, we can conclude that UBE2O probably modifies protein with various ubiquitination manners including monoubiquitination, K48-linked ubiquitination and K63-linked ubiquitination.

In terms of the biological functions, UBE2O is originally believed to participate in erythroid differentiation because it is highly expressed in reticulocytes and can ubiquitinate endogenous proteins involved in erythroid cells [2, 3]. Recent evidence shows that UBE2O is involved in adipogenesis [4], actin polymerization [25], inflammation [7], nuclear trafficking of chromatin-associated proteins [5], and tumorigenesis [6]. Different from previous findings, the present study suggests that UBE2O acts as a negative modulator in MM because it is downregulated in MM cells which was confirmed by both DNA microarray and RT-PCR. When it is restored, UBE2O induces MM cell apoptosis and delays MM tumor growth in nude mice (Fig. 6), suggesting UBE2O is probably a tumor suppressor protein against MM. This action of UBE2O suppressing MM is highly associated with c-Maf expression, c-Maf protein ubiquitination, and degradation because UBE2O fails to induce apoptosis of MM cells lacking c-Maf. c-Maf is a frequently expressed basic leucine zipper transcription factors in MM. As a transcription factor, c-Maf binds to the Maf recognition element (MARE) of downstream genes and triggers their transcription. These downstream genes mainly include cyclin D2, integrin β7, CCR1, and ARK5, which are responsible for cell cycle progress, bone marrow stromal cell adhesion, MM cell proliferation, and myeloma cell invasion, respectively [10]. However, out of expectation, UBE2O fails to arrest MM cell cycles at the G0/G1phase. This finding probably suggests that UBE2O also targets other proteins that also modulate MM cell cycle and cell proliferation, but these proteins act on other cell cycle phases, such as S or G2/M phases.

In the present study, we also found that UBE2O is downregulated in MM cells, how does this happens is not clear. The probable mechanisms include suppression of gene transcription modulated by small interfering RNA or non-coding RNA or other factors in gene expression regulation. In addition, c-Maf is a basic zipper family transcription factor and this class of transcription factors displays various regulations in gene transcription, including both promotion and suppression cell differentiation or cell proliferation [8]. Whether UBE2O is negatively modulated by c-Maf is not known; it will be very interesting if UBE2O-targeted therapy towards MM can be developed.

The present study showed that UBE2O inhibits proliferation and induces apoptosis of MM cells expressing c-Maf but not those lacking c-Maf, because MM is highly heterogeneous. Our previous studies showed that the c-Maf status is a significant factor for MM cell response to dexamethasone, a mainstay of MM treatment [21]. This study showed that targeting UBE2O could be a promising strategy for the treatment of a subset of c-Maf expressing MM.

## Conclusions

Taken together, the present study identifies that UBE2O as a novel regulator modulates c-Maf protein stability by mediating its polyubiquitination and subsequent degradation in proteasomes. UBE2O specifically induces apoptosis and inhibits proliferation of a subset of MM cells that express c-Maf. This study provides a novel insight in understanding c-Maf biological function and targeted MM therapy.

## Abbreviations

7-ADD: 7-Aminoactinomycin D
AP/MS: Affinity purification-coupled mass spectrometry
BAP1: BRCA1 associated protein-1
CCND2: Cyclin D2
CCR1: C-C chemokine receptor type 1
GEO: Gene Expression Omnibus
IP: Immunoprecipitation
KEGG: Kyoto Encyclopedia of Genes and Genomes
LC/MS/MS: Liquid chromatography tandem mass spectrometry
MARE: c-Maf recognition element
MGUS: Monoclonal gammopathy of undetermined significance
MM: Multiple myeloma
MTT: 3-(4,5-Dimethylthiazol-2-yl)-2,5-diphenyltetrazolium bromide
PI: Propidium iodide
SMM: Smoldering multiple myeloma
TRAF6: TNF receptor associated factor 6
UBE2O: Ubiquitin-conjugating enzyme E2 O
